# Supplementary material for: B-Cell Epitopes-Based Chimeric Protein from SARS-CoV-2 N and S Proteins Is Recognized by Specific Antibodies in Serum and Urine Samples from Patients
Source: Viruses. 2023 Sep 5;15(9):1877. doi: 10.3390/v15091877 (PMC10538162; doi:10.3390/v15091877)
Supplement: Supplementary file 1 [file viruses-15-01877-s001.zip › viruses-2588888-supplementary.pdf]

SUPPLEMENTARY FIGURES

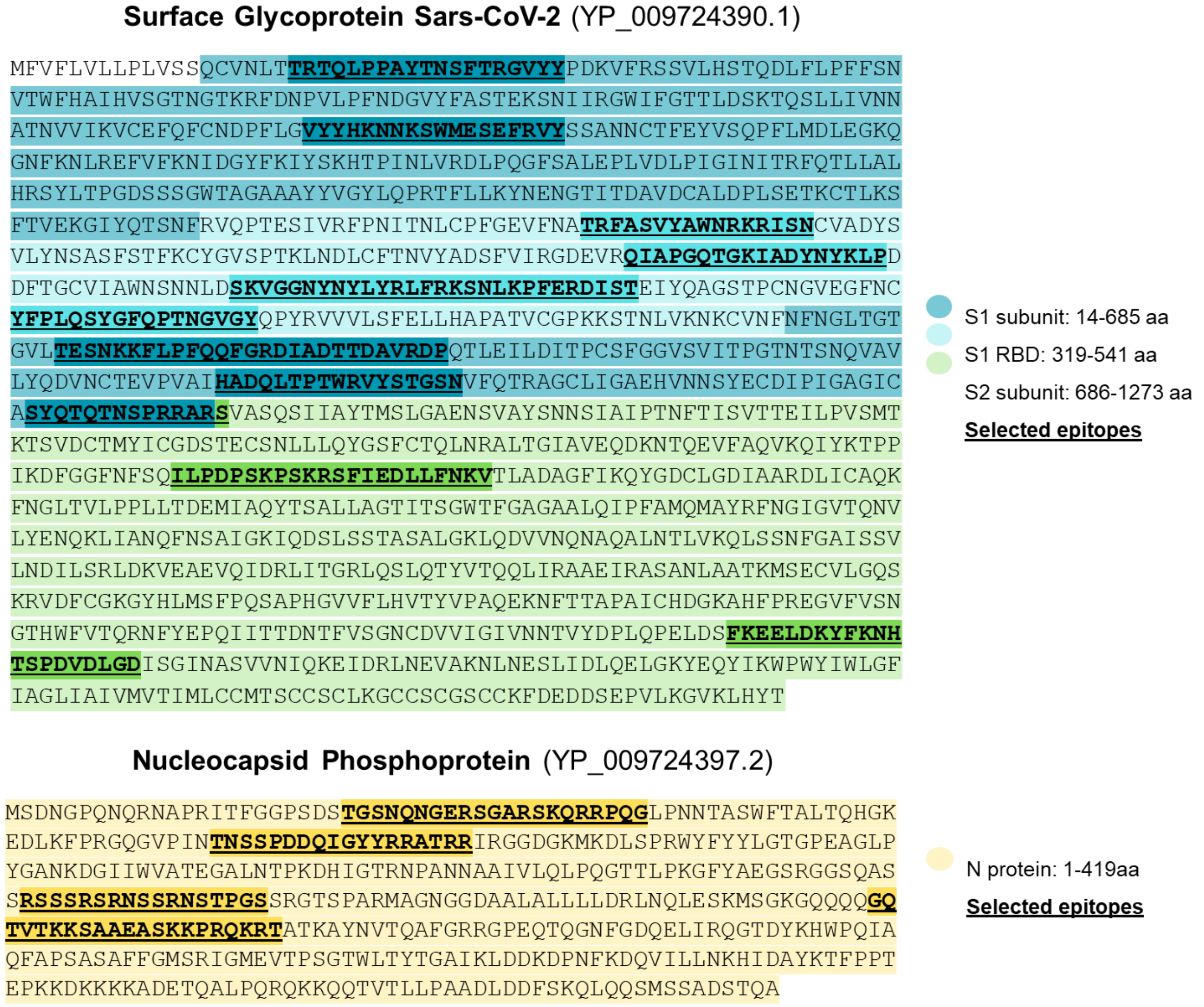

Supplementary Figure S1. Identification of linear B-cell epitopes by bioinformatics analysis. The amino acid sequences of surface glycoprotein (YP_009724390.1) and nucleocapsid phosphoprotein (YP_009724397.2) proteins were evaluated by bioinformatics tools. Four linear B-cell epitopes were selected from N protein and eleven from S protein.

Surface glycoprotein

```
Sars-CoV-2      --MFVFLVLLPLVS-------------------SQCVNLTTRTQLPPAYTNSFTRGVYYP
Sars-CoV-1      --MFIFLLFLTLTSGS---------------DLDRCTTFDDVQAPNYTQHTSSMRGVYYP
HCoV-229E       --MFVLLVA---------------------------------------------------
HCoV-NL63       MKLFLILLVLPLASCFFTCNSNANLSMLQLGVPDNSSTIVTGLLPTHWFCANQSTSVYSA
HCoV-OC43       --MFLILLISLPTAFAVIGDLNCPLDPKLKGSFNNRDTGSPSISTDTVD-VTNGLGTYYV
HCoV-HKU1       ---MLLIIFILPTTLAVIGDFNCTNF-----AINDKNTTVPRISEYVVD-VSYGLGTYYI
MERS-CoV        MIHSVFLLMFLLTPTESYVDVGPDSVKSACIEVDIQQTFFDKTWPRPID-VSKADGIIYP
                    ::::

Sars-CoV-2      DKVFRSSVLHSTQDLFLPF---FSNVTWFHAIHVSGTNGTKRF----DNPVLPFNDGVYF
Sars-CoV-1      DEIFRSDTLYLTQDLFLPF---YSNVTGFHTINHTFGNP-----------VIPFKDGIYF
HCoV-229E       ----------------------YALLHIAGCQTTNGTN----------------------
HCoV-NL63       NGFFYIDVGNHRSAFALHTGYYDANQYYIYVTNEIGLNASVTL------KICKFSRNTTF
HCoV-OC43       LDRVYLNTTLFLNGYYPTSGSTYRNMALKGTDLLSTLWFKPPF-------LSDFINGIFA
HCoV-HKU1       LDRVYLNTSILFTGYFPKSGANFRDLSLKGTTYLSTLWYQKPF-------LSDFNNGIFS
MERS-CoV        QGRTYSNITITYQGLF-PYQGDHGDMYVYSAGHATGTTPQKLFVANYSQDVKQFANGFVV

Sars-CoV-2      ASTEKSN-----------------IIRGWIFGTTLDSKT---------QSL---LIVNNA
Sars-CoV-1      AATEKSN-----------------VVRGWVFGSTMNNKS---------QSV---IIINNS
HCoV-229E       ------------------------------------------------------------
HCoV-NL63       DFLSNASSSFDCIVN---------LLFTEQLGAPLGITISGETVRLHLYNVTRTFYVPAA
HCoV-OC43       KVKNTKVFKDGVMYS---------EFPAITIGSTFVNTS---------YSV---VVQPRT
HCoV-HKU1       RVKNTKLYVNKTLYS---------EFSTIVIGSVFINNS---------YTI---VVQPHN
MERS-CoV        RIGAAANSTGTVIISPSTSATIRKIYPAFMLGSSVGNFSDGKMGRFFNHTL---VLLPDG

Sars-CoV-2      TNV----------VIKV-------------------CEFQFCND----------PFLGVY
Sars-CoV-1      TNV----------VIRA-------------------CNFELCDN----------PFFAVS
HCoV-229E       ------------------------------------TSHSVCNGCVGHSENVFAVESGGY
HCoV-NL63       YKLTKLSVKCYFNYSCVFSVVNATVTVNVTTHNGRVVNYTVCDDCNGYTDNIFSVQQDGR
HCoV-OC43       INSTQDGVNKLQGLLEV-----------------SVCQYNMCEH----------PHTICH
HCoV-HKU1       ------------GVLEI-----------------TACQYTMCEY----------PHTICK
MERS-CoV        CGT----------LLRAFYCILEPRSGNHCPAGNSHTSFATYHT----------PATDCS
                                                     ..

Sars-CoV-2      YHKNN---KSWMESEFRVYSSANNCTFEY---VSQPFLMDLEGKQGNFKNLREFVFKNID
Sars-CoV-1      KPMGT-------QTHTMIFDNAFNCTFEY---ISDAFSLDVSEKSGNFKHLREFVFKNKD
HCoV-229E       IPSNFAFNNWFLLTNTSSVVDGVVRSFQ-------PLLLNCLWSVSGSQFTTGFVYFNGT
HCoV-NL63       IPNGFPFNNWFLLTNGSTLVDGVSRLYQ-------PLRLTCLWPVPGLKSSTGFVYFNAT
HCoV-OC43       PNLGNHFKELW-HLDTGVVSCLYKRNFTY------DVNATYLY----------FHFYQEG
HCoV-HKU1       SK-GSFRNESW-HFDKSEPLCLFKKNFTY------NVSTDWLY----------FHFYQER
MERS-CoV        D--GNYNRNAS-LNSFKEYFNLRNCTFMYTYNITEDEILEWFGITQTAQGVHLFSSR---
                   .                      :                          *

Sars-CoV-2      GY---FKIYSKHTPINLVRDLPQGFSALEPLVDLPIGINI--TRFQTLLALHR-SYLTPG
Sars-CoV-1      GF---LYVYKGYQPIDVVRDLPSGFNTLKPIFKLPLGINI--TNFRAILTAFS------P
HCoV-229E       GRGA-CKGFYSNASSDVIR-----YNIN-FEENLRRGTILFKTSYGAVVFYCTNNTLVSG
HCoV-NL63       GSDVNCNGYQHNSVVDVMR-YNLNFSAN-SLDNLKSGVIVFKTLQYDVLFYCSNSSSGVL
HCoV-OC43       GT---FYAYFTDT----------GFVTK-FLFNVYLGMAL--SHYYVMPLTCI------S
HCoV-HKU1       GT---FYAYYADS----------GMPTT-FLFSLYLGTLL--SHYYVLPLTCNAISSNTD
MERS-CoV        -Y---VDLYGG----NMFQ-----FATL-PVYD------T--IKYYSIIPHSI-RSIQSD
                        :                       .              :

Sars-CoV-2      DSSSGWTAGAAAYYVGYLQPRTFLLKYNENGTITDAVDCALDPLSETKCTLKSFTVEK-G
Sars-CoV-1      AQDI-WGTSAAAYFVGYLKPTTFMLKYDENGTITDAVDCSQNPLAELKCSVKSFEIDK-G
HCoV-229E       DAHIPSGTVLGNFYCF------------VNTTIGN--ETTSAFVGALPKTVREFVISRTG
HCoV-NL63       DTTIPFGPSSQPYYCF------------INSTINT--THVSTFVGILPPTVREIVVARTG
HCoV-OC43       RRDIGF---TLEYWVTPLTPRQYLLAFNQDGIIFNAVDCMSDFMSEIKCKTQSIAPPT-G
HCoV-HKU1       NETLQY-------WVTPLSKRQYLLKFDNRGVITNAVDCSSSFFSEIQCKTKSLLPNT-G
MERS-CoV        RKA--W----AAFYVYKLQPLTFLLDFSVDGYIRRAIDCGFNDLSQLHCSYESFDVES-G
                             :                  *          ..    .  .:      *

Sars-CoV-2      IYQTSNFRVQPTESIVRFPNITNLCPFGEVFNATRFASVYAWNRKRISNCVADYS-----
```

```
Sars-CoV-1      IYQTSNFRVVPSGDVVRFPNITNLCPFGEVFNATKFPSVYAWERKKISNCVADYS-----
HCoV-229E       HFYINGYRYFSLGNV-------EAVNFNVTNAATTVCT---VALASYADVLVNVSQTAIA
HCoV-NL63       QFYINGFKYFDLGFI-------EAVNFN--VTTASATDFWTVAFATFVDVLVNVSATNIQ
HCoV-OC43       VYELNGYTVQPIADVYRRKPDLPNCNIEAWLNDKSVPSPLNWERKTFSNCNFNMS-----
HCoV-HKU1       VYDLSGFTVKPVATVHRRIPDLPDCDIDKWLNNFNVPSPLNWGRKIFSNCNFNLS-----
MERS-CoV        VYSVSSFEAKPSGSVVEQAEGVE-CDFSPLLSGTP-PQVYNFKRLVFTNCNYNLT-----
                 :  ..:       :           :                     :   : :

Sars-CoV-2      -VLYNSASFSTFKCYGVSPTKLNDLCFTNVYADSFVIRGDEVR-QIAPGQTGKIADYNYK
Sars-CoV-1      -VLYNSTFFSTFKCYGVSATKLNDLCFSNVYADSFVVKGDDVR-QIAPGQTGVIADYNYK
HCoV-229E       NIIYCNSVINRLRCDQLSFDVPDG-FYSTSPIQPVELPVSIVSLPVYHKHTFIVLYVNFE
HCoV-NL63       NLLYCDSPFEKLQCEHLQFGLQDG-FYSANFLDDNVLPETYVALPIYYQHT----DINFT
HCoV-OC43       -SLMSFIQADSFTCNNIDAAKIYGMCFSSITIDKFAIPNRRKV-DLQLGNLGYLQSSNYR
HCoV-HKU1       -TLLRLVHTDSFSCNNFDESKIYGSCFKSIVLDKFAIPNSRRS-DLQLGSSGFLQSSNYK
MERS-CoV        -KLLSLFSVNDFTCSQISPAAIASNCYSSLILDYFSYPLSMKS-DLSVSSAGPISQFNYK

                  :      . : *  ..     .  :.    :            :           *:

Sars-CoV-2      LPDDFTGCVIAWNSNNLDSKVGG----NY-NYLYRLFRKS--------------------
Sars-CoV-1      LPDDFMGCVLAWNTRNIDATSTG----NY-NYKYRYLRHG--------------------
HCoV-229E       HQRGPGKC-YNCRPAVINITLANFNETKG-PLCVDTSHFT-----------------TQF
HCoV-NL63       ATASFGGSCYVCKPHQVNISLNG----NT-SVCVRTSHFS-----------------IRY
HCoV-OC43       IDTTATSCQLYYNLPAANVSVSRFNPSTW-NKRFGFIEDSVFVPQPTGVFTNHSVVYAQH
HCoV-HKU1       IDTTSSSCQLYYSLPAINVTINNYNPSSW-NRRYGFNNFN---------LSSHSVVYSRY
MERS-CoV        QSFSNPTCLILATVPHNLTTITKPLKYSYINKCSRLLSDD--------------------
                       .           .       .

Sars-CoV-2      ------------------NLKPFERDISTEIYQAGSTPCNGV---------------EGF
Sars-CoV-1      ------------------KLRPFERDISNVPFSPDGKPCTPP----------------AL
HCoV-229E       VDNVK------------LARWSASINTGNCPFSFG-----------------------KV
HCoV-NL63       IYNRVKSGSPG------DSSWHIYLKSGTCPFSFS-----------------------KL
HCoV-OC43       CFKAPKNFCPC------SSCPGKNNGIGTCPAGTNSLTCDNL------------CTLDPI
HCoV-HKU1       CFSVNNTFCPCAKPSFASSCKSHKPPSASCPIGTNYRSCESTTVLDHTDWCRCSCLPDPI
MERS-CoV        -----------------------RTEVPQLVNANQYSPCVSI---------------VPS


Sars-CoV-2      NCYFPLQSYGFQPTN--GVGYQPYRVVV-----------------------LSFE----L
Sars-CoV-1      NCYWPLNDYGFYTTT--GIGYQPYRVVV-----------------------LSFE----L
HCoV-229E       INFVKFGSVCFSLKYIPGGCAMPIMANLV-----------NHKSHNIGSLYVSWSDGDVI
HCoV-NL63       NNFQKFKTICFSTVEVPGSCNFPLEATWH-----------YTSYTIVGALYVTWSEGNSI
HCoV-OC43       TFKAPDTYKCPQTKSLVGIGEHCSGLAVKSDYCG------NNSCTCQPQAFLGWSADSCL
HCoV-HKU1       TAYDPRS--CSQKKSLVGVGEHCAGFGVDEEKCGVLDGSYNVSCLCSTDAFLGWSYDTCV
MERS-CoV        TVWEDGDYYRKQLSPLEGGGWLVAS---------------GSTVAMTEQLQMGFGITVQY
                                 *                                 : :

Sars-CoV-2      LHAPATVC----------GPKKSTNLVKNK-------CVNFNFNGLTGTGVLTESNKKFL
Sars-CoV-1      LNAPATVC----------GPKLSTDLIKNQ-------CVNFNFNGLTGTGVLTPSSKRFQ
HCoV-229E       TGVPKPVE----------GVSSFMNVTLNK-------CTKYNIYDVSGVGVIRISNDTFL
HCoV-NL63       TGVPYPVS----------GIREFSNLVLNN-------CTKYNIYDYVGTGIIRSSNQSLA
HCoV-OC43       QGDKCNIFANFILHDVNNGLTCSTDLQKANTEIELGVCVNYDLYGISGQGIFVEVNATYY
HCoV-HKU1       SNNRCNIFSNFILNGINSGTTCSNDLLQSNTEVFTDVCADYDLYGITGQGIFKEVSAVYY
MERS-CoV        GTDTNSVCP---------KLEFANDTKIAS---QLGNCVEYSLYGVSGRGVFQNCTAVGV
                      :                 :    .       *..:.: .  * *::   .

Sars-CoV-2      P-FQQFGRDI-ADTTDAVRDPQTLEILDITPCSFGGVSVITPGTNTSNQVAVLYQDVNCT
Sars-CoV-1      P-FQQFGRDV-SDFTDSVRDPKTSEILDISPCSFGGVSVITPGTNASSEVAVLYQDVNCT
HCoV-229E       N-GITYTSTS-GNLLG-FKDVTNGTIYSITPCNPPDQLVVYQQAVVGAMLSENFTSYG--
HCoV-NL63       G-GITYVSNS-GNLLG-FKNVSTGNIFIVTPCNQPDQVAVYQQSIIGAMTAVNESRYG--
HCoV-OC43       NSWQNLLYDSNGNLYG-FRDYITNRTFMIHSCYSGRVSAAYHANSPEP--ALLFRNIKCN
HCoV-HKU1       NSWQNLLYDSNGNIIG-FKDFVTNKTYNIFPCYAGRVSAAFHQNASSL--ALLYRNLKCS
MERS-CoV        R-QQRFVYDAYQNLVGYYSD--DGNYYCLRACVSVPVSVIYDKETKTH--ATLFGSVACE
                            :  .   :        : .*      .           :

Sars-CoV-2      EVPVAI--HADQLTPTWRVYSTGSNVFQTRAGCLIGAEHVNNSY---ECDIPIGAGICAS
Sars-CoV-1      DVSTAI--HADQLTPAWRIYSTGNNVFQTQAGCLIGAEHVDTSY---ECDIPIGAGICAS
HCoV-229E       --------FSN-VVEMPKFFYASNGTY----NCTDAV-LTYSSF-----------GVCAD
HCoV-NL63       --------LQN-LLQLPNFYYVSNGGN----NCTTAV-MTYSNF-----------GICAD
HCoV-OC43       YV------FNNSLTRQLQPINYSFDSY---LGCVVNA-YNSTAISVQTCDLTVGSGYCVD
HCoV-HKU1       YV------LNN-ISLATQPY---FDSY---LGCVFNA-DNLTDYSVSSCALRMGSGFCVD
```

```
MERS-CoV        HISSTMSQYSRSTRSMLKRRDSTYGPLQTPVGCVLG--LVNSSLFVEDCKLPLGQSLCAL

                                 .      .      .*        .             . *.

Sars-CoV-2      YQTQ--TNSPRRARSVASQ---SIIAYTMSLGAENSVAY--SNNSIAIPTNFTISVTTEI
Sars-CoV-1      YHTVS-LLRSTSQKSI--------VAYTMSLGADSSIAY--SNNTIAIPTNFSISITTEV
HCoV-229E       G-----SIIAVQPRNVSYD------------SVSAIVTA-----NLSIPSNWTTSVQVEY
HCoV-NL63       G-----SLIPVRPRNSSDN------------GISAIITA-----NLSIPSNWTTSVQVEY
HCoV-OC43       Y-----SKNRRSRRAITTGYRFTNFEPFTVNSVNDSLEPVGGLYEIQIPSEFTIGNMEEF
HCoV-HKU1       YNSPSSSSSRRKRRSISASYRFVTFEPFNVSFVNDSIESVGGLYEIKIPTNFTIVGQEEF
MERS-CoV        PDTPS-TLTPRSVRSVPGEMRLASIAFNHPIQVDQLNSS---YFKLSIPTNFSFGVTQEY
                             .                   .           : **::::     *

Sars-CoV-2      LPVSMTKTSVDCTMYICGDSTECSNLLLQYGSFCTQLNRALTGIAVEQDKNTQEVFAQVK
Sars-CoV-1      MPVSMAKTSVDCNMYICGDSTECANLLLQYGSFCTQLNRALSGIAAEQDRNTREVFAQVK
HCoV-229E       LQITSTPIVVDCSTYVCNGNVRCVELLKQYTSACKTIEDALRNSAMLESADVSEMLTFDK
HCoV-NL63       LQITSTPIVVDCATYVCNGNPRCKNLLKQYTSACKTIEDALRLSAHLETNDVSSMLTFDS
HCoV-OC43       IQTSSPKVTIDCAAFVCGDYAACKLQLVEYGSFCDNINAILTEVNELLDTTQLQVANSLM
HCoV-HKU1       IQTNSPKVTIDCSLFVCSNYAACHDLLSEYGTFCDNINSILDEVNGLLDTTQLHVADTLM
MERS-CoV        IQTTIQKVTVDCKQYVCNGFQKCEQLLREYGQFCSKINQALHGANLRQDDSVRNLFASVK
                :  .     :**  ::*..   *   * :*   *  ::  *             :

Sars-CoV-2      Q-IYKTPPIKDFGGF-----NFS-QILP----DPSKPSKRSFIEDLLFNKVTLADAGFIK
Sars-CoV-1      Q-MYKTPTLKYFGGF-----NFS-QILP----DPLKPTKRSFIEDLLFNKVTLADAGFMK
HCoV-229E       K-AFTLANVSSFGDY-----NLS-SVIPSLPRSGSRVAGRSAIEDILFSKLVTSGLGTVD
HCoV-NL63       N-AFSLANVTSFGDY-----NLS-SVLPQRNIRSSRIAGRSALEDLLFSKVVTSGLGTVD
HCoV-OC43       NGVTLSTKLKDGVNFNVDDINFS-PVLGCLGSECSKASSRSAIEDLLFDKVKLSDVGFVE
HCoV-HKU1       QGVTLSSNLNTNLHFDVDNINFK-SLVGCLGPHCGS-SSRSFFEDLLFDKVKLSDVGFVE
MERS-CoV        S-SQSSPIIPGFGG------DFNLTLLEPVSISTGSRSARSAIEDLLFDKVTIADPGYMQ
                .     . :           ::.  ::          : ** :**:**.*:  :. * :.

Sars-CoV-2      Q-YGDCL--GDIAARDLICAQKFNGLTVLPPLLTDEMIAQYTSALLAGTITSGWTFGAGA
Sars-CoV-1      Q-YGECL--GDINARDLICAQKFNGLTVLPPLLTDDMIAAYTAALVSGTATAGWTFGAGA
HCoV-229E       ADYKKCT--KGLSIADLACAQYYNGIMVLPGVADAERMAMYTGSLIGGIALGGLTSAAS-
HCoV-NL63       VDYKSCT--KGLSIADLACAQYYNGIMVLPGVADAERMAMYTGSLIGGMVLGGLTSAAA-
HCoV-OC43       A-YNNCT--GGAEIRDLICVQSYKGIKVLPPLLSENQISGYTLAATSASLFPPWTAAAG-
HCoV-HKU1       A-YNNCT--GGSEIRDLLCVQSFNGIKVLPPILSESQISGYTTAATVAAMFPPWSAAAG-
MERS-CoV        G-YDDCMQQGPASARDLICAQYVAGYKVLPPLMDVNMEAAYTSSLLGSIAGVGWTAGLSS
                  * .*         ** *.*   *  *** :   .  : ** :   .      : . .

Sars-CoV-2      ALQIPFAMQMAYRFNGIGVTQNVLYENQKLIANQFNSAIGKIQDSLSSTAS---------
Sars-CoV-1      ALQIPFAMQMAYRFNGIGVTQNVLYENQKQIANQFNKAISQIQESLTTTST---------
HCoV-229E       ---IPFSLAIQSRLNYVALQTDVLQENQRILAASFNKAMTNIVDAFTGVNDAITQTSQAL
HCoV-NL63       ---IPFSLALQARLNYVALQTDVLQENQKILAASFNKAINNIVASFSSVNDAITQTAEAI
HCoV-OC43       ---VPFYLNVQYRINGLGVTMDVLSQNQKLIANAFNNALHAIQQGFDATNS---------
HCoV-HKU1       ---IPFSLNVQYRINGLGVTMDVLNKNQKLIATAFNNALLSIQNGFSATNS---------
MERS-CoV        FAAIPFAQSIFYRLNGVGITQQVLSENQKLIANKFNQALGAMQTGFTTTNE---------
                   :**   :  *:* :.:  :** :**. :*  **.*:  :  .:  .

Sars-CoV-2      -----ALGKLQDVVNQNAQALNTLVKQLSSNFGAISSVLNDILSRLDKVEAEVQIDRLIT
Sars-CoV-1      -----ALGKLQDVVNQNAQALNTLVKQLSSNFGAISSVLNDILSRLDKVEAEVQIDRLIT
HCoV-229E       QTVATALNKIQDVVNQQGNSLNHLTSQLRQNFQAISSSIQAIYDRLDIIQADQQVDRLIT
HCoV-NL63       HTVTIALNKIQDVVNQQGSALNHLTSQLRHNFQAISNSIQAIYDRLDSIQADQQVDRLIT
HCoV-OC43       -----ALVKIQAVVNANSEALNNLLQQLSNRFGAISASLQEILSRLDALEAEAQIDRLIN
HCoV-HKU1       -----ALAKIQSVVNSNAQALNSLLQQLFNKFGAISSSLQEILSRLDALEAQVQIDRLIN
MERS-CoV        -----AFRKVQDAVNNNAQALSKLASELSNTFGAISASIGDIIQRLDVLEQDAQIDRLIN
                     *: *:* .** :..:*. * .:*   * ***  :  * .*** :: : *:****.

Sars-CoV-2      GRLQSLQTYVTQQLIRAAEIRASANLAATKMSECVLGQSKRVDFCGKGYHLMSFPQSAPH
Sars-CoV-1      GRLQSLQTYVTQQLIRAAEIRASANLAATKMSECVLGQSKRVDFCGKGYHLMSFPQAAPH
HCoV-229E       GRLAALNVFVSHTLTKYTEVRASRQLAQQKVNECVKSQSKRYGFCGNGTHIFSLVNAAPE
HCoV-NL63       GRLAALNAFVSQVLNKYTEVRGSRRLAQQKINECVKSQSNRYGFCGNGTHIFSIVNSAPD
HCoV-OC43       GRLTALNAYVSQQLSDSTLVKFSAAQAMEKVNECVKSQSSRINFCGNGNHIISLVQNAPY
HCoV-HKU1       GRLTALNAYVSQQLSDISLVKFGAALAMEKVNECVKSQSPRINFCGNGNHILSLVQNAPY
MERS-CoV        GRLTTLNAFVAQQLVRSESAALSAQLAKDKVNECVKAQSKRSGFCGQGTHIVSFVVNAPN
                *** :*:.:*:: *         .   *  *:.*** .** * .***:* *:.*:   **

Sars-CoV-2      GVVFLHVTYVPAQEKNFTTAPAICHDG---KAHFPREGVFVSNGTH-----WFVTQRNFY
Sars-CoV-1      GVVFLHVTYVPSQERNFTTAPAICHEG---KAYFPREGVFVFNGTS-----WFITQRNFF
```

```
HCoV-229E        GLVFLHTVLLPTQYKDVEAWSGLCVDGI--NGYVLRQPNLALYKEGNY---YRITSRIMF
HCoV-NL63        GLLFLHTVLLPTDYKNVKAWSGICVDGI--YGYVLRQPNLVLYSDNGV---FRVTSRVMF
HCoV-OC43        GLYFIHFNYVPTKYVTAKVSPGLCIAGN--RGIAPKSGYFVNVNNT-----WMYTGSGYY
HCoV-HKU1        GLLFMHFSYKPISFKTVLVSPGLCISGD--VGIAPKQGYFIKHNDH-----WMFTGSSYY
MERS-CoV         GLYFMHVGYYPSNHIEVVSAYGLCDAANPTNCIAPVNGYFIKTNNTRIVDEWSYTGSSFY
                 *: *:*    * .        .:*  .         .  :           :  *    :

Sars-CoV-2       EPQIITTDNTFVSGNCDVVIGIVNNTVYDPLQPELDS-------FKEELDKYFKNHTSPD
Sars-CoV-1       SPQIITTDNTFVSGNCDVVIGIINNTVYDPLQPELDS-------FKEELDKYFKNHTSPD
HCoV-229E        EPRIPTIADFVQIENCNVTFVNISRSELQTIVPEYIDVNKTLQELSYKLPNYTVPDLVVE
HCoV-NL63        QPRLPVLSDFVQIYNCNVTFVNISRVELHTVIPDYVDVNKTLQEFAQNLPKYVKPNFDLT
HCoV-OC43        YPEPITENNVVVMSTCAVNYTKAPYVMLNTSIPNLPD-------FKEELGQWFKNQTSVA
HCoV-HKU1        YPEPISDKNVVFMNTCSVNFTKAPLVYLNHSVPKLSD-------FESELSHWFKNQTSIA
MERS-CoV         APEPITSLNTKYVAP-QVTYQNISTNLPPPLLGNSTGI-----DFQDELDEFFKNVSTSI
                  *      :        *               .  .       :  :*  :

Sars-CoV-2       V------DLG-DISGINASVVNIQKEIDRLNEVAKNLNESLIDLQELGKYEQYIKWPWYI
Sars-CoV-1       V------DLG-DISGINASVVNIQKEIDRLNEVAKNLNESLIDLQELGKYEQYIKWPWYV
HCoV-229E        QYNQTILNLTSEISTLENKSAELNYTVQKLQTLIDNINSTLVDLKWLNRVETYIKWPWWV
HCoV-NL63        PFNLTYLNLSSELKQLEAKTASLFQTTVELQGLIDQINSTYVDLKLLNRFENYIKWPWWV
HCoV-OC43        P------DLSLDY--INVTFLDLQVEMNRLQEAIKVLNHSYINLKDIGTYEYYVKWPWYV
HCoV-HKU1        P------NLTLNFHTINATFLDLYYEMNLIQESIKSLNNSYINLKDIGTYEMYVKWPWYV
MERS-CoV         P------NFG-SLTQINTTLLDLTYEMLSLQQVVKALNESYIDLKELGNYTYYNKWPWYI
                        ::  .   :: .  .:      ::   . :* : ::*: :.    * ****::

Sars-CoV-2       WLGFIAGLIAIVMVTIMLCCMTSCCSCLKG-CCSCGSCC-----KFDEDDSEPVLKGVKL
Sars-CoV-1       WLGFIAGLIAIVMVTILLCCMTSCCSCLKG-ACSCGSCC-----KFDEDDSEPVLKGVKL
HCoV-229E        WLCISVVLIFVVSMLLLCCCSTGCCGFFSCFASSIRGCCE--STKLPYYDVEKI------
HCoV-NL63        WLIISVVFVVLLSLLVFCCLSTGCCGCCNCLTSSMRGCCDCGSTKLPYYEFEKV------
HCoV-OC43        WLLICLAGVAMLVLLFFICCCTGCGTSCFK---KCGGCCD----DYTGYQELVIKTS---
HCoV-HKU1        WLLISFSFIIFLVLLFFICCCTGCGSACFS---KCHNCCD----EYGGHHDFVIKTS---
MERS-CoV         WLGFIAGLVALALCVFFILCCTGCGTNCMG-KLKCNRCCD----RYEEYDLEP-------
                 ** :    : .    .:    *.*         .   **

Sars-CoV-2       HYT
Sars-CoV-1       HYT
HCoV-229E        HIQ
HCoV-NL63        HVQ
HCoV-OC43        HDD
HCoV-HKU1        HDD
MERS-CoV         HKV
                 *
```

Nucleocapsid Phosphoprotein

```
SARS-CoV-2       -MSDNGPQ-NQRNAPR-IT---------FGGPSDSTGSNQNGERSGARSKQRRPQ-----
SARS-CoV-1       -MSDNGPQSNQRSAPR-IT---------FGGPTDSTDNNQNGGRNGARPKQRRPQ-----
HCoV-OC43        MSFTPGKQSSSRASSGNRSGNGILK---WADQSDQFRNVQTRG------RRAQPKQTSTS
HCoV-HKU1        MSYTPGHHAGSRSSSGNRS--GILKKTSWVDQSERSHQTYNRG------RKPQPKFTVST
HCoV-229E        --------------MATVK---------WADASEP-----QRG------RQGRIP-----
HCoV-NL63        --------------MASVN---------WADDRAA--------------RKKFPP-----
MERS-CoV         --------MASPAAPRAVS---------FADNNDITNTNLSRGRG----RNPKPR-----
                                   .         : .                  ..

SARS-CoV-2       -----GLPNNTASWFTALTQHGKE-DLKFPRGQGVPINTNSSPDDQIGYYRRATRR-IRG
SARS-CoV-1       -----GLPNNTASWFTALTQHGKE-ELRFPRGQGVPINTNSGPDDQIGYYRRATRR-VRG
HCoV-OC43        QQPLGGNVVPYYSWFSGITQFQKGKEFEFAEGQGVPIAPGVPATEAKGYWYRHNRRSFKT
HCoV-HKU1        QPQ--GNPIPHYSWFSGITQFQKGRDFKFPDGQGVPIAYGIPPSEAKGYWYKHNRRSFKT
HCoV-229E        -----------YSLYSPLLVDSEQ-PWKVIPRNLVPVNKK-DKNKLIGYWNVQKR--FRT
HCoV-NL63        -----------PSFYMPLLVSSDKAPYRVIPRNLVPIGKG-NKDEQIGYWNVQER--WRM
 MERS-CoV         -----AAPNNTVSWYTGLTQHGKV-PLTFPPGQGVPLNANSTPAQNAGYWRRQDRK-INT
                            * :  :    .     .   : **:       .  **:    *   .

SARS-CoV-2       GDGKMKDLSPRWYFYYLGTGPEAGLPYGANKDGIIWVATEGALNTPKDHIGTRNPANNAA
SARS-CoV-1       GDGKMKELSPRWYFYYLGTGPEASLPYGANKEGIVWVATEGALNTPKDHIGTRNPNNNAA
```

```
HCoV-OC43       ADGNQRQLLPRWYFYYLGTGPHAKDQYGTDIDGVFWVASNQADVNTPADIVDRDPSSDEA
HCoV-HKU1       ADGQQKQLLPRWYFYYLGTGPYASSSYGDAHEGIFWVASHQADTSIPSDVSARDPTIQEA
HCoV-229E       RKGKRVDLSPKLHFYYLGTGPHKDAKFRERVEGVVWVAVDGAKTEP-TGYGVRRKNSEPE
HCoV-NL63       RRGQRVDLPPKVHFYYLGTGPHKDLKFRQRSDGVVWVAKEGAKTVN-TSLGNRKRNQKPL
MERS-CoV        GNG-IKQLAPRWYFYYTGTGPEAALPFRAVKDGIVWVHEYGATDAP-STFGTRNPNNDSA
                  *   :* *. :*** ****     :    :*:.**    *          *    .

SARS-CoV-2      IV---LQLPQGTTLPKGFYAEGSRGGSQASSRSSSR--SRNSSRNSTPGSSRGTSPARMA
SARS-CoV-1      TV---LQLPQGTTLPKGFYAEGSRGGSQASSRSSSR--SRGNSRNSTPGSSRGNSPARMA
HCoV-OC43       IP---TRFPPGTVLPQGYYIEGSG-------RSAPN--SRSTSRTSSRAFSAGS--RSRA
HCoV-HKU1       IP---TRFSPGTILPQGYYVEGSG-------RSASN--SRPGSRSQSRGPNNRS--LSRS
HCoV-229E       IPHFNQKLPNGVTVAE---EPDSRAPSRSQSRSQSR--SRGESKSQSRNPSSDR--NHNS
HCoV-NL63       EPKFSIALPPELSVVE--FEDRSNNSSRASSRSSTRNNSRDSSRSTSRQQSRTRSDSNQS
MERS-CoV        IV---TQFAPGTKLPKNFHIEGTGGNSQSSSRASSV--SRNSSRSSSQGSRSGN--STRG
                       :.    : :      :        *: .   **  *.. :            .

SARS-CoV-2      GNGGDAALALLL---------LDRLNQLESKMSGKGQQQQG-------------QTVTKK
SARS-CoV-1      SGGGETALALLL---------LDRLNQLESKVSGKGQQQQG-------------QTVTKK
HCoV-OC43       SSGNRAPTSGVIPDMADQ---IASLVLAKLGKDATKPQQVT-------------KHTAKE
HCoV-HKU1       NSNFRHSDSIVKPDMADE---IASLVLAKLGKD-SKPQQVT-------------KQNAKE
HCoV-229E       QDDIMKAVAAALKSLGFDKPQEKDKKSAKTGTPKPSRNQSPASSQSAAKILARSQSSETK
HCoV-NL63       SSDLVAAVTLALKNLGFD---NQSKSPSSSGTSTPKKPNKP-------------LSQPRS
MERS-CoV        TSPGPSGIGAVGGDLLY----LDLLNRLQALESGKVKQSQP-------------KVITKK
                 .                          .         .                    .

SARS-CoV-2      SAAEASKKPRQKRTATKA--YNVTQAFGRRGPEQTQGNFGDQELIRQGTDYKHWPQIAQF
SARS-CoV-1      SAAEASKKPRQKRTATKQ--YNVTQAFGRRGPEQTQGNFGDQDLIRQGTDYKHWPQIAQF
HCoV-OC43       VRQKILNKPRQKRSPNKQ--CTVQQCFGKRGPNQ---NFGGGEMLKLGTSDPQFPILAEL
HCoV-HKU1       IRHKILMKPRQKRTPNKF--CNVQQCFGKRGPLQ---NFGNSEMLKLGTNDPQFPILAEL
HCoV-229E       EQKHEMQKPRWKRQPNDDVTSNVTQCFGPRDLDH---NFGSAGVVANGVKAKGYPQFAEL
HCoV-NL63       DKPSQLKKPRWKRVPTRE--ENVIQCFGPRDFNH---NMGDSDLVQNGVDAKGFPQLAEL
MERS-CoV        DAAAAKNKMRHKRTSTKS--FNMVQAFGLRGPGDLQGNFGDLQLNKLGTEDPRWPQIAEL
                       * *.** ..     .: *.** *.      *:*.  :   *..   :* :*::

SARS-CoV-2      APSASAFFGMSRIGMEVTPS-----------GTWLTYTGAIKLDDKDPNFKDQVILLNKH
SARS-CoV-1      APSASAFFGMSRIGMEVTPS-----------GTWLTYHGAIKLDDKDPQFKDNVILLNKH
HCoV-OC43       APTAGAFFFGSRLELAKVQNLSGNPDEPQKDVYELRYNGAIRFDSTLSGFETIMKVLNEN
HCoV-HKU1       APTPGAFFFGSKLELFKRDS---DADSPSKDTFELRYSGSIRFDSTLPGFETIMKVLKEN
HCoV-229E       VPSTAAMLFDSHIVSKESGN-----------TVVLTFTTRVTVPKDHPHLGKFL----EE
HCoV-NL63       IPNQAALFFDSEVSTDEVGD-----------NVQITYTYKMLVAKDNKNLPKFI----EQ
MERS-CoV        APTASAFMGMSQFKLTHQNN-----DDHGNPVYFLRYSGAIKLDPKNPNYNKWLELLEQN
                 *. .*::  * .      .              : :   : .          :    :

SARS-CoV-2      IDAY-----------KTFPPTEPK--KDKKKKADETQALPQRQKKQQTVTLLPAADLDDF
SARS-CoV-1      IDAY-----------KTFPPTEPK--KDKKKKTDEAQPLPQRQKKQPTVTLLPAADMDDF
HCoV-OC43       LNAY---QQQDGMMNMSPKPQRQRGLKNGQGENDNISVASPKSRVQQNKSRELTAEDISL
HCoV-HKU1       LDAYVNSNQNTVSGSLSPKPQRKRGVKQSPESFDSLNLSADTQHISN----DFTPEDHSL
HCoV-229E       LNAF------------------TREMQQQPLLNPSALEFNPSQTSPATV--EPVRDEVSI
HCoV-NL63       ISAF-------------TKPSSIKEMQSQSSHVAQNTVLNASIPES-----KPLADDDSA
MERS-CoV        IDAY-----------KTFPKKEKKQKAPKEESTDQMSEPPKEQRVQGSIT-QRTRTRPSV
                :.*:                   .          .                       .

SARS-CoV-2      SKQLQQSMS--SADSTQA
SARS-CoV-1      SRQLQNSMSGASADSTQA
HCoV-OC43       LKKMDEPY---TEDTSEI
HCoV-HKU1       LATLDDPY---VEDSVA-
HCoV-229E       ETDI-------IDEVN--
HCoV-NL63       IIEI-------VNEVLH-
MERS-CoV        QPGP-------MIDVNTD
                             :
```

Supplementary Figure S2: Amino acid sequence alignment of N4S11-CoV2 and some other CoV virus of interest. Alignment was conducted by the CLUSTAL multiple sequence alignment / MUSCLE (3.8). Legend: Asterix (*) positions with a single, fully conserved residue. Colon (:) positions with conservation between amino acid groups of similar properties. Period (.) positions with conservation between amino acid groups of weakly similar properties. S protein accession numbers: YP_009724390.1 Surface glycoprotein [SARS-CoV-2], P59594.1 Spike glycoprotein

[SARS-CoV], K9N5Q8.1 Spike glycoprotein [MERS-CoV], QNT54842.1 surface glycoprotein [Human coronavirus 229E], AIX10763.1 Spike glycoprotein [Human coronavirus OC43], YP_003767.1 spike protein [Human coronavirus NL63], AXT92555.1 spike glycoprotein [Human coronavirus HKU1]. N protein accession number: YP_009724397.2C Nucleocapsid phosphoprotein [SARS-CoV-2], P59595.1 Nucleocapsid protein [SARS-CoV], K9N4V7.1 Nucleocapsid protein [MERS-CoV], WDE18042.1 surface glycoprotein [Human coronavirus 229E], QDH43730.1 N [Human coronavirus OC43], QED88044.1 nucleocapsid protein [Human coronavirus NL63], AYN64565.1 nucleocapsid phosphoprotein [Human coronavirus HKU1].

Sars-CoV- 2 Variants	Pep 1 - S1 subunit	Pep 2 - S1 subunit	Pep 4 - RBD S1 subunit	Pep 5 - RBD S1 subunit	Pep 7 - RBD S1 subunit
N4S11-SC2	TRTQLPPAYTNSFTRGVYY	VYYHKNNKSWMESEFRVY	TRFASVYAWNRKRISN	QIAPGQTGKIADYNYKLP	SKVGGNYNYLYRLFRKSNLKPFERDIST
Alpha	TRTQLPPAYTNSFTRGVYY	VYYHKNNKSWMESEFRVY	TRFASVYAWNRKRISN	QIAPGQTGKIADYNYKLP	SKVGGNYNYLYRLFRKSNLKPFERDIST
Beta	TRTQLPPAYTNSFTRGVYY	VYYHKNNKSWMESEFRVY	TRFASVYAWNRKRISN	QIAPGQTGNIADYNYKLP	SKVGGNYNYLYRLFRKSNLKPFERDIST
Gamma	NRTQLPSAYTNSFTRGVYY	VYYHKNNKSWMESEFRVY	TRFASVYAWNRKRISN	QIAPGQTGKIADYNYKLP	SKVGGNYNYLYRLFRKSNLKPFERDIST
Delta	TRTQLPPAYTNSFTRGVYY	VYYHKNNKSWMESEFRVY	TRFASVYAWNRKRISN	QIAPGQTGKIADYNYKLP	SKVGGNYNYLYRLFRKSNLKPFERDIST
Omicron	TRTQLPPAYTNSFTRGVYY	VYYHKNNKSWMESEFRVY	TRFASVYAWNRKRISN	QIAPGQTGNIADYNYKLP	SKVSGNYNYLYRLFRKSNLKPFERDIST

Sars-CoV- 2 Variants	Pep 8 - RBD S1 subunit	Pep 9 - S1 subunit	Pep 10 - S1 subunit	Pep 11 - S1 subunit	Pep 13 - S2 subunit
N4S11-SC2	YFPLQSYGFQPTNGVGY	TESNKKFLPFQQFGRDIADTTDAVRDP	HADQLTPTWRVYSTGSN	SYQTQTNSPRRARS	ILPDPSKPSKRSFIEDLLFNKV
Alpha	YFPLQSYGFQPTYGVGY	TESNKKFLPFQQFGRDIADTTDAVRDP	HADQLTPTWRVYSTGSN	SYQTQTNSHRRARS	ILPDPSKPSKRSFIEDLLFNKV
Beta	YFPLQSYGFQPTYGVGY	TESNKKFLPFQQFGRDIADTTDAVRDP	HADQLTPTWRVYSTGSN	SYQTQTNSPRRARS	ILPDPSKPSKRSFIEDLLFNKV
Gamma	YFPLQSYGFQPTYGVGY	TESNKKFLPFQQFGRDIADTTDAVRDP	HADQLTPTWRVYSTGSN	SYQTQTNSPRRARS	ILPDPSKPSKRSFIEDLLFNKV
Delta	YFPLQSYGFQPTNGVGY	TESNKKFLPFQQFGRDIADTTDAVRDP	HADQLTPTWRVYSTGSN	SYQTQTNSPRRARS	ILPDPSKPSKRSFIEDLLFNKV
Omicron	YFPLRSYSFRPTYGVGY	TESNKKFLPFQQFGRDIADTTDAVRDP	HADQLTPTWRVYSTGSN	SYQTQTKSHRRARS	ILPDPSKPSKRSFIEDLLFNKV

Sars-CoV- 2 Variants	Pep 15 - S2 subunit	N Protein	N Protein	N Protein (CTD)	N Protein (NTD)
N4S11-SC2	FKEELDKYFKNHTSPDVDLGD	TGSNQNGERSGARSKQRRPQG	RSSSRSRNSSRNSTPGS	GQTVTKKSAAEASKKPRQKRT	TNSSPDDQIGYYRRATRR
Alpha	FKEELDKYFKNHTSPDVDLGD	TGSNQNGERSGARSKQRRPQG	RSSSRSRNSSRNSTPGS	GQTVTKKSAAEASKKPRQKRT	TNSSPDDQIGYYRRATRR
Beta	FKEELDKYFKNHTSPDVDLGD	TGSNQNGERSGARSKQRRPQG	RSSSRSRNSSRNSTPGS	GQTVTKKSAAEASKKPRQKRT	TNSSPDDQIGYYRRATRR
Gamma	FKEELDKYFKNHTSPDVDLGD	TGSNQNGERSGARSKQRRPQG	RSSSRSRNSSRNSTPGS	GQTVTKKSAAEASKKPRQKRT	TNSSRDDQIGYYRRATRR
Delta	FKEELDKYFKNHTSPDVDLGD	TGSNQNGERSGARSKQRRPQG	RSSSRSRNSLRNSTPGS	GQTVTKKSAAEASKKPRQKRT	TNSSPDDQIGYYRRATRR
Omicron	FKEELDKYFKNHTSPDVDLGD	TGSNQNGERSGARSKQRRPQG	RSSSRSRNSSRNSTPGS	GQTVTKKSAAEASKKPRQKRT	TNSSPDDQIGYYRRATRR

Supplementary Figure S3. Comparative analysis of amino acid sequences of selected peptides in N4S11-CoV2 and some SARS-2 variants of interest. Surface glycoprotein - N4S11-SC2 Peptides (Wuhan-Hu-1, YP_009724390.1); Alpha (B.1.1.7, QWE88920.1); Beta (B.1.351, UPD30382.1); Gamma (P.1, QVQ47339.1); Delta (B.1.617.2, UHV13980.1); and Omicron (UOX09450.1). Nucleocapsid protein - N4S11-SC02 Peptides (Wuhan-Hu-1, YP_009724397.2); Alpha (B.1.1.7, QWE88928.1); Beta (B.1.351, UPD30390.1); Gamma (P.1, QVQ47347.1); Delta (B.1.617.2, UHV13987.1); Omicron (UOX09458.1) (Sanches et al., 2021; Alcantara et al., 2022). Red color indicates amino acid variation.

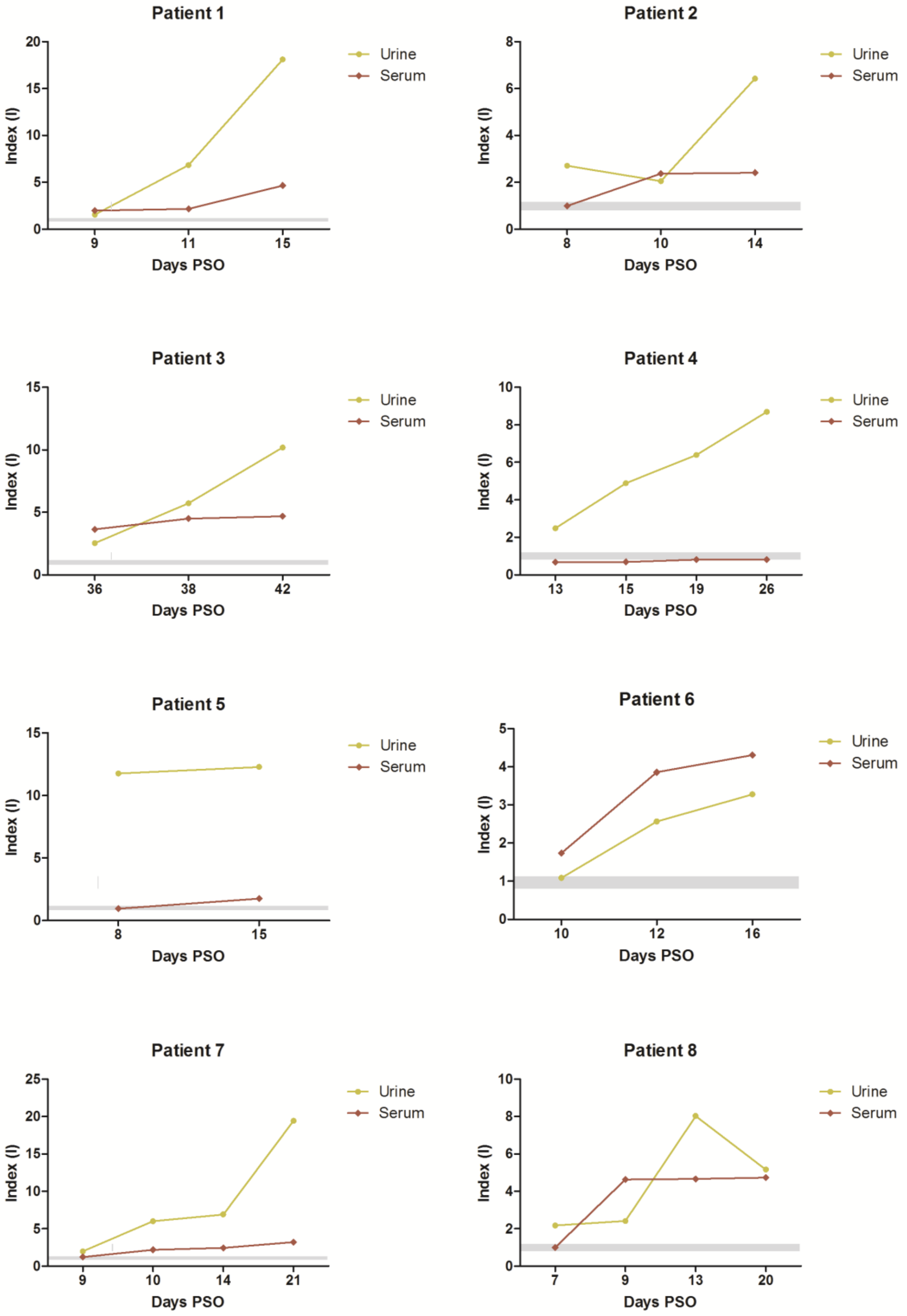

Supplementary Figure S4. Dynamics of IgG antibody conversion in patient urine and serum samples. Figures show the IgG levels specific to the N4S11-SC2 for eight patients, with longitudinal collection on different days post-symptom onset. The plotted index values (I) were related to the absorbance ratio on the cut-off.
